# Factors affecting pouch-related outcomes after restorative proctocolectomy

**DOI:** 10.1371/journal.pone.0186596

**Published:** 2017-10-19

**Authors:** Gyoung Tae Noh, Jeonghee Han, Min Soo Cho, Hyuk Hur, Byung Soh Min, Kang Young Lee, Nam Kyu Kim

**Affiliations:** Department of Surgery, Yonsei University College of Medicine, Seoul, South Korea; Kurume University School of Medicine, JAPAN

## Abstract

**Purposes:**

Restorative proctocolectomy (RPC) with ileal pouch anal anastomosis (IPAA) is the procedure of choice for patients with familial adenomatous polyposis (FAP) and ulcerative colitis (UC) despite morbidities that can lead to pouch failure. We aimed to identify factors associated with pouch-related morbidities.

**Methods:**

A retrospective analysis of patients who underwent RPC with IPAA was performed. To investigate the factors associated with pouch-related morbidities, patients' preoperative demographic and clinical factors, and intraoperative factors were included in the analysis.

**Results:**

A total of 49 patients with UC, FAP, and colorectal cancer were included. Twenty patients (40.8%) experienced leakage-related, functional, and/or pouchitis-related morbidities. Patients with American Society of Anesthesiologists (ASA) grade 2 or 3 had a higher risk of functional morbidity than those with grade 1. Intraoperative blood loss exceeding 300.0 mL was associated with an increased risk of pouchitis-related morbidity.

**Conclusions:**

Our study demonstrated associations of higher ASA grade and increased intraoperative blood loss with poor functional outcomes and pouchitis, respectively.

## Introduction

Restorative proctocolectomy (RPC) with ileal pouch anal anastomosis (IPAA) is the procedure of choice for patients with familial adenomatous polyposis (FAP) and ulcerative colitis (UC), and it is performed in selected cases of dysplasia or carcinoma of the colon or rectum [1, 2]. Since Parks and Nicholls first described an S-shaped IPAA as an ileal pouch with a hand-sewn anastomosis to the dentate line after mucosectomy of the remnant rectum, this procedure has evolved with the introduction of the J pouch and stapled anastomosis in the 1980s [3, 4]. The shift in the surgical paradigm to minimally invasive surgery led to the introduction of a laparoscopic approach to RPC, which had initially been performed via an open approach and accompanied diverting ileostomy. This shift resulted in fewer long-term complications and even the avoidance of diverting ileostomy in select cases [5–7].

RPC with IPAA completely removes the diseased colonic and rectal mucosa while maintaining normal sphincter function and avoiding the morbidity associated with permanent ileostomy; accordingly, most patients obtain satisfactory results [8–10]. However, IPAA remains a technically demanding procedure with relatively high perioperative morbidity rates ranging from 30% to 60%, and some patients experience pouch failure and eventual pouch excision [11–15]. Pouch failure after IPAA has been reported to occur in 5–10% of patients due to anastomotic complications, pouch dysfunction, recalcitrant pouchitis, and other emergency presentations such as bowel ischemia and obstruction [14, 16–18].

Preoperative identification of patients at risk for pouch-related morbidities would facilitate the establishment of surgical strategies and allow better counseling and consideration of alternative surgical treatment options for diseases otherwise amenable to IPAA. Furthermore, increased recognition of the intraoperative factors associated with pouch-related morbidities could enable the avoidance of these factors. In this context, we reviewed the characteristics of our patients and investigated pouch-related morbidities after RPC with IPAA to identify morbidity-associated factors. We further compared the outcomes between surgical methods to determine the optimal surgical options.

## Materials and methods

The medical records of consecutive patients who underwent RPC with IPAA for the treatment of FAP, UC, and colorectal cancer between January 2006 and December 2014 were reviewed retrospectively. The study was reviewed and approved by the Severance Hospital Institutional Review Board. A waiver of informed consent was approved by the Severance Hospital Institutional Review Board given the retrospective nature of the study. The operations were performed by 5 surgeons, and all patients in the stated period underwent total proctocolectomy and IPAA simultaneously in an elective setting. Surgery was performed through either an open or a laparoscopic approach, and the choice of hand-sewn or stapled anastomosis was determined according to the degree of rectal inflammation in patients with UC and the severity of rectal polyposis in those with FAP. An ileal pouch was created using a linear stapler intracorporeally or extracorporeally, according to surgeon preference. The configuration of the ileal pouch was J-shaped with a length of 20 cm, which was identical in both the laparoscopic and open approach. For hand-sewn IPAA, mucosectomy of the lower rectum was performed routinely through a perineal approach. For stapled IPAA, double-stapled anastomosis without mucosectomy was performed with the staple line in the very low rectum, and the rectal cuff was shortened as much as technically possible. Protective loop ileostomy was optional. Decisions regarding ileostomy formation were made intraoperatively and based on the technical difficulty of anastomosis, mesenteric tension, intraoperative primary impermeability of the anastomosis at control, and the patient’s preference.

Preoperative patient data included age, sex, underlying disease, American Society of Anesthesiologists (ASA) grade, body mass index (BMI), smoking history, and the presence of malignancy. Operative data included surgical approach (open or laparoscopic), method of anastomosis (hand-sewn or stapled), formation of diverting ileostomy, duration of the operation, blood loss during the operation, and intraoperative transfusion. Postoperative data included 3 types of pouch-related morbidities: pouch-leakage-related, pouchitis-related, and pouch function-related complications.

Demographic and clinical variables were defined as follows. The presence of malignancy was defined as the coexistence of pathologically diagnosed colonic or rectal adenocarcinoma. Pouch leakage was defined as a defect in the anastomosis or pouch stump, and pouch-associated fistula was defined as an abnormal passage or sinus from the pouch to another surface or organ. Pouchitis was defined as clinical presentation with typical symptoms of pouchitis (increased number and looser consistency of bowel movements, rectal bleeding, urgency, incontinence, and/or abdominal or pelvic cramping) and at least 1 abnormal pouch endoscopy during a symptomatic episode. Pouchitis-related stricture was defined as the appearance of narrowing at the anastomosis that required surgical dilation during the follow-up period for pouchitis. Intractable diarrhea was defined as antidiarrheal agent-unresponsive stool frequency more than 10 times a day for more than 1 year without evidence of infection. Fecal incontinence was defined as a prolonged involuntary loss of liquid or solid stool that required pad use for more than 1 year after surgery. Pouch failure was defined as the need for permanent stoma construction with excision of the ileoanal pouch or abdominoperineal reconstruction for complications.

Data were analyzed using SPSS Statistics (version 20.0; IBM Corp., Armonk, NY, USA). Descriptive results are presented as medians and interquartile ranges (Q1–Q3) for continuous outcomes, and as frequencies and percentages for categorical outcomes. A binary logistic regression model was used to identify risk factors for pouch-related morbidities. Variables with a *P*-value < 0.10 in the univariate analysis were selected for multivariate analysis. Continuous variables were dichotomized according to clinical implications or by using the median value of each variable as the cut-off value. To compare the outcomes between surgical methods, continuous variables were analyzed using a Mann-Whitney U test, and categorical variables were analyzed using a chi-square test. A *P*-value < 0.05 was considered statistically significant.

## Results

### Patient characteristics

A total of 49 patients were included in the analysis, and the median follow-up period was 41.0 months (24.0–82.5 months). The characteristics of all enrolled patients are summarized in Table 1. The median patient age was 40.0 years, and the sex distribution was almost even (25 male and 24 female patients). The mean BMI was 22.0 kg/m^2^ (19.0–24.0 kg/m^2^), and 9 patients (18.4%) had a history of smoking.

**Table 1:** Preoperative and operative characteristics.

Demographics	
Age (years)	40.0 (31.0–51.0)
Sex	
Male	25 (51.0%)
Female	24 (49.0%)
Underlying disease	
Ulcerative colitis	31 (63.3%)
Familial adenomatous polyposis	16 (32.7%)
Colorectal cancer	2 (4.1%)
^†^ASA classification	
Grade 1	25 (51.0%)
Grade 2	18 (36.7%)
Grade 3	6 (12.2%)
^‡^BMI (kg/m^2^)	22.0 (19.0–24.0)
Smoking history	
Present	9 (18.4%)
Absent	40 (81.6%)
Presence of malignancy	
Present	12 (24.5%)
Absent	37 (75.5%)
Surgical modality	
Open approach	15 (30.6%)
Laparoscopic approach	34 (69.4%)
Anastomosis method	
Hand-sewn	34 (69.4%)
Double-stapled	15 (30.6%)
Diverting ileostomy	
Present	40 (81.6%)
Absent	9 (18.4%)
Duration of operation (minutes)	332.0 (280.0–438.0)
Blood loss during operation (mL)	150.0 (25.0–475.0)
Intraoperative transfusion	
Present	9 (18.4%)
Absent	40 (81.6%)

Data are presented as medians (interquartile range, Q1-Q3), or n (%).

^†^ASA: American Society of Anesthesiologists.

^‡^BMI: body mass index.

Thirty-one patients (63.3%) had been diagnosed with UC, and 16 (32.7%) had been diagnosed with FAP. In addition, 2 patients had been diagnosed with colorectal cancer without underlying UC or FAP; one patient had triple synchronous cancers that arose from the ascending colon, rectosigmoid junction, and distal rectum, and the other was diagnosed with a solitary rectal cancer that caused complete rectal obstruction. Twenty-five (51.0%), 18 (36.7%), and 6 patients (12.2%) were classified with ASA grade 1, 2, and 3, respectively. Combined malignancies that originated from the colorectal mucosa were identified pathologically in 12 patients (24.5%).

Thirty-four (69.4%) and 15 patients (30.6%) underwent surgery via a laparoscopic approach and open approach, respectively. In 34 patients (69.4%), hand-sewn ileoanal anastomoses were performed, while stapled anastomoses were performed in 15 patients (30.6%). For the laparoscopic approach, 22 patients (64.7%) and 12 patients (35.3%) underwent hand-sewn and stapled anastomoses, respectively. For the open approach, 12 patients (80.0%) and 3 patients (20.0%) underwent hand-sewn and stapled anastomoses, respectively. There was no significant relationship between surgical approach and anastomosis method (*P* = 0.336). Diverting ileostomy was performed in 40 patients (81.6%). The median duration of the operation was 332.0 minutes (280.0–438.0 minutes). The median blood loss volume was 150.0 mL (25.0–475.0 mL), and 9 patients (18.4%) received intraoperative transfusion.

### Pouch-related morbidity

Twenty patients (40.8%) experienced pouch-related morbidities (Table 2). Pouch leakage-related morbidity occurred in 8 patients (16.8%), including 6 patients (16.3%) with pouch leakage and 2 patients (4.1%) with a fistula between the pouch and the vagina. Pouchitis was observed in 8 patients (16.3%), and 2 (4.1%) experienced anastomotic stricture. Eight patients (16.3%) experienced intractable diarrhea for more than 1 year after surgery, and 3 (6.1%) presented with fecal incontinence. Two patients (4.1%) with pouch failure required pouch excision because of pouch leakage and pouch-vaginal fistula, respectively. Comparing the incidence of pouch-related morbidities according to the surgeon groups categorized by surgical experience, 38 patients (77.6%) operated by two surgeons with experience of more than 10 cases showed 36.8% and 11 patients (22.4%) operated by the other three surgeons with experience of less than 10 cases showed 54.5%, which was not statistically significant (*P* = 0.320).

**Table 2:** Pouch-related morbidity profiles.

Variables	Number (%)
Overall morbidity	20 (40.8)
Leakage related	8 (16.3)
Pouch leakage	6 (12.2)
Pouch associated fistula	2 (4.1)
^†^Pouchitis related	8 (16.3)
Pouchitis	8 (16.3)
Pouchitis related stricture	2 (4.1)
^†^Function related	8 (16.3)
Intractable diarrhea (>10 times/day)	8 (16.3)
Fecal incontinence	3 (6.1)

^†^Data include duplicate patients.

### Factors associated with pouch-related morbidities

The results of the analysis for the associations between the pre-/intraoperative factors and these morbidities are described in Table 3. Patients with ASA grade 2 and 3 had a higher risk of function-related morbidity relative to those with grade 1 (odds ratio [OR] = 9.9; 95% confidence interval [CI]: 1.1–87.9; *P* = 0.04). Intraoperative blood loss exceeding 300.0 mL was associated with an increased risk of pouchitis (OR = 7.3; 95% CI: 1.3–43.1; *P* = 0.025). Furthermore, patients who received an intraoperative blood transfusion had an increased risk of pouchitis-related morbidities (OR = 7.2; 95% CI: 1.4–38.3; *P* = 0.021). No statistically significant associations were observed with other preoperative factors, including underlying disease entity (UC or FAP), BMI > 25 kg/m^2^, smoking history, and presence of malignancy, or with surgical factors, such as the surgical approach (open or laparoscopic), method of anastomosis (hand-sewn or stapled), or formation of a diverting ileostomy. In the multivariate analysis, patients who experienced intraoperative blood loss exceeding 300.0 mL exhibited a significantly increased risk of pouchitis-related morbidities (OR = 6.3; 95% CI: 1.0–38.9; *P* = 0.047). Although not statistically significant, an association between intraoperative blood transfusion and a trend towards an increased risk of pouchitis (OR = 6.1; 95% CI: 1.0–36.8; *P* = 0.05) was seen.

**Table 3:** Factors associated with pouch-related morbidities.

Variables	Leakage related morbidity		Pouchitis related morbidity		Function related morbidity	
	OR (95% CI)	*P*	OR (95% CI)	*P*	OR (95% CI)	*P*
Age (over 40 years)	1.930 (0.407–9.160)	0.408	1.930 (0.107–9.160)	0.408	3.833 (0.690–21.302)	0.125
Sex (female)	1.930 (0.407–9.160)	0.408	0.571 (0.120–2.711)	0.481	0.571 (0.120–2.711)	0.481
Underlying disease						
Familial adenomatous polyposis	1	0.256	1	0.187	1	0.556
Ulcerative colitis	3.6 (0.394–32.871)		4.375 (0.488–39.184)		1.680 (0.298–9.466)	
^†^ASA grade (grade 2, 3)	1.050 (0.231–4.778)	0.95	1.930 (0.407–9.160)	0.408	9.882 (1.111–87.902)	0.04
^‡^BMI (over 25 kg/m^2^)	1.944 (0.315–11.996)	0.474	N/A		1.733 (0.073–6.568)	0.750
Smoking history	N/A		1.619 (0.269–9.748)	0.599	0.589 (0.063–5.497)	0.643
Presence of malignancy	1.033 (0.179–5.958)	0.971	N/A		N/A	
Surgical modality						
Open approach	1	0.707	1	0.204	1	0.204
Laparoscopic approach	1.393 (0.247–7.858)		0.367 (0.078–1.725)		0.367 (0.078–1.725)	
Anastomosis method						
Hand-sewn	1	0.707	1	0.654	1	0.247
Double-stapled	0.718 (0.127–4.051)		1.450 (0.298–7.051)		0.276 (0.031–2.468)	
Diverting ileostomy	0.618 (0.103–3.719)	0.599	N/A		1.697 (0.182–15.831)	0.643
Duration of operation (> 332.0 minute)	3.474 (0.626–19.283)	0.154	0.952 (0.209–4.334)	0.95	0.261 (0.047–1.450)	0.125
Blood loss during operation (>300.0 mL)	3.590 (0.743–17.346)	0.112	7.250 (1.278–41.139)	0.025	0.521 (0.093–2.905)	0.457
Intraoperative transfusion	1.619 (0.269–9.748)	0.599	7.200 (1.353–38.326)	0.021	3.5 (0.657–18.648)	0.142

^†^ASA: American Society of Anesthesiologists.

^‡^BMI: body mass index.

### Subgroup analysis according to surgical method

Table 4 presents a comparison of outcomes, including the duration of the operation, intraoperative blood loss volume, duration of postoperative hospital stay, and the occurrence of pouch-related morbidity, according to the surgical methods. In comparison with open surgery, laparoscopic surgery showed a longer operative duration, reduced blood loss, and a shortened postoperative hospital stay, which were not statistically significant. Both methods were associated with similar rates of pouch-related morbidity. Similar results were achieved with comparisons of hand-sewn vs. double-stapled anastomosis, and the performance or omission of diverting ileostomy.

**Table 4:** Factors associated with pouch-related morbidities.

Variables	Surgical modality			Anastomosis method			Diverting ileostomy		
	Open	Laparoscopy	*P*	Hand-sewn	Double-stapled	*P*	Present	Absent	*P*
Duration of operation (minutes)	299.0	353.5	0.140	322.0	353.0	0.428	324.0	353.0	0.423
	(243.0–459.0)	(306.0–437.5)		(270.0–437.5)	(321.0–459.0)		(273.3–436.0)	(318.0–471.0)	
Blood loss during operation (mL)	220.0	150.0	0.463	210.0	100.0	0.120	200.0	100.0	0.162
	(0.0–550.0)	(37.5–412.5)		(50.0–500.0)	(0.0–100.0)		(50.0–200.0)	(0.0–100.0)	
Postoperative hospital stay (days)	12.0	11.5	0.306	11.0	12.0	0.281	11.5	12.0	0.326
	(11.0–14.0)	(8.0–15.25)		(9.0–13.3)	(11.0–17.0)		(9.0–13.8)	(10.5–17.5)	
Pouch related morbidity									
Present	4 (26.7%)	9 (26.5%)	1.000	9 (26.5%)	4 (26.7%)	1.000	11 (27.5%)	2 (22.2%)	1.000
Absent	11 (73.3%)	25 (73.5%)		25 (73.5%)	11 (73.3%)		29 (72.5%)	7 (77.8%)	

## Discussion

In this study, 40.8% of patients developed pouch-related morbidities after RPC with IPAA. In addition, 4.1% of patients experienced pouch failure that required pouch excision. These high complication rates underscore the technical complexity of RPC with IPAA and indicate the substantial problems encountered by surgeons. According to previous studies, perioperative morbidity after RPC with IPAA ranges from 30% to 60% [11–15], with reported pouch failure rates of 3.5–15% [14, 16, 19–22]. In previous reports, pouch-related morbidities have been described as pouch-pelvic sepsis, including pouch-sacral, -perineal, or -vaginal fistulas or anastomotic defects, pouchitis, and pouch dysfunction [18, 23–26]. We categorized our patients using similar criteria for leakage-related, pouchitis-related, and function-related morbidities and analyzed the data to identify the factors associated with pouch-related morbidity.

In our analysis, higher ASA grade and increased blood loss during the operation were found to be significantly relevant to function-related and pouchitis-related morbidities, respectively. Patients with ASA grades 2 or 3 were more likely to develop function-related morbidities, such as intractable diarrhea or fecal incontinence, compared to those with ASA grade 1. This result is generally acceptable because patients in poor general condition would be expected to have poor remnant bowel or anal function. Patients who experienced blood loss during the operation in excess of 300.0 mL were found to have an increased risk of pouchitis compared to those with less blood loss, and a trend towards an increased risk for pouchitis was observed in patients who received an intraoperative blood transfusion, although this increase was not statistically significant. In a previous study of predictive factors for pouchitis, the authors reported that intraoperative transfusion was associated with the development of pouchitis, and described the role of hypoxemia in the development of pouchitis [27]. Although the importance of systemic hypoxemia in the pathogenesis of pouchitis has not been described and requires further investigation, hypovolemia and inadequate perfusion caused by intraoperative blood loss might induce hypoxemia in the pouch.

Previous reports have suggested that underlying disease entity and severity, as well as obesity, affect pouch-related morbidities [16, 28, 29]. Fazio et al. [16] reported that UC increased the risk of stricture and pouchitis in a study of surgical outcomes and quality of life after IPAA in 3,707 patients. In another previous report, multivariate analysis revealed that patients with pancolitis were at risk of developing chronic pouchitis [28]. In a recent study, the authors suggested that obesity, defined as a BMI > 30, was an independent risk factor for pouch-related complications, citing technical complexity and challenges associated with IPAA creation in obese patients as reasons [29]. Taken together, these risk factors indicate that particularly careful follow-up is required for patients after RPC with IPAA because of the high likelihood of pouch-related morbidities leading to pouch failure and a need for reoperation.

Regarding surgical methods for RPC with IPAA, several studies have reported poor outcomes with hand-sewn anastomosis. For example, Manilich et al. [30] suggested that hand-sewn anastomosis was associated with a higher ileal pouch failure rate, compared with stapled anastomosis. Additionally, an association between hand-sewn anastomosis and pouch failure was reported by MacRae et al [31]. In contrast, a meta-analysis of 4,183 patients failed to show a significant effect of hand-sewn anastomosis on pouch survival [32]. In our study, the anastomosis method was not significantly associated with pouch-related morbidities. Furthermore, no significant differences in other surgical outcomes were observed between hand-sewn and stapled anastomosis.

In the present study, the comparison of open and laparoscopic approaches also failed to reveal significant differences in surgical outcomes. The choice of an open vs. laparoscopic approach is no longer a controversial issue. Laparoscopic approach has replaced the open approach with the development of technology and accumulation of experience with minimally invasive surgery. In the context of RPC with IPAA, laparoscopic surgery is considered both safe and feasible, which has been advocated by multiple studies [33]. In the present study, the proportion of laparoscopic approach to RPC with IPAA was 69.4, and this is expected to increase in the future.

Along with the expansion of the laparoscopic approach for RPC with IPAA, attempts have been made to skip fecal diversion. Traditionally, diverting ileostomy was routinely performed after IPAA. However, the role of the diverting ileostomy is currently controversial. Recently, Kiran et al. reported that a proximal diverting ileostomy did not protect against pelvic sepsis [34], and other studies have supported the finding that proximal diversion does not prevent anastomotic leakage [35, 36]. In accordance with those results, the present study found no significant association between the formation of a diverting ileostomy and pouch-related morbidity.

We must acknowledge the limitations of the present study. First, this was a retrospective study and not a prospective trial. Therefore, uncontrollable and unknown biases, including recall bias, information bias, and selection bias, may have been present. Untested variables that may have influenced the outcome could include the number of surgeons and physicians. Second, few centers that perform IPAA will experience a large number of pouch-related morbidities. As such, this study involved a single-center retrospective series and was inherently limited by a small sample size and relatively short follow-up period. Given these limitations, the results of this study cannot be considered confirmative evidence, and must be considered the basis for future studies. However, this study has provided a comprehensive analysis of potential risk factors for pouch-related morbidities and the impact of surgical methods on surgical outcomes. Certainly, further studies will be required to advance our knowledge of the surgical consequences of RPC with IPAA in order to better manage such patients.

In conclusion, the present study found associations between a higher ASA grade and a poor functional outcome, and between increased intraoperative blood loss and the occurrence of pouchitis. However, surgical method-related factors, such as the surgical approach (laparoscopic or open), anastomosis method (hand-sewn or stapled), and the omission of a diverting ileostomy, did not affect surgical outcomes.

## Supporting information

S1 Dataset Data for enrolled patients underwent restorative proctocolectomy with ileal pouch anal anastomosis. (XLSX)
